# Bacterial Extracellular DNA Production Is Associated with Outcome of Prosthetic Joint Infections

**DOI:** 10.1155/2018/1067413

**Published:** 2018-10-22

**Authors:** Beata Zatorska, Carla Renata Arciola, Nicolas Haffner, Luigi Segagni Lusignani, Elisabeth Presterl, Magda Diab-Elschahawi

**Affiliations:** ^1^Department of Infection Control and Hospital Epidemiology, Medical University of Vienna, Waehringer Guertel 18-20, 1090 Vienna, Austria; ^2^Department of Experimental, Diagnostic and Specialty Medicine, University of Bologna, Italy; ^3^Laboratory on Implant Infections, IRCCS Rizzoli Orthopaedic Institute of Bologna, Bologna, Italy; ^4^Department of Orthopedics and Traumatology, Hanusch Hospital, Heinrich-Collin-Str. 30 1140 Vienna, Austria; ^5^Department of Infection Control and Hospital Epidemiology, 2 Medical University of Vienna, Waehringer Guertel 18-20, 1090 Vienna, Austria

## Abstract

In a retrospective study the association of the production of extracellular DNA (eDNA) in biofilms of clinical staphylococcal isolates from 60 patients with prosthetic joint infection (PJI) and the clinical outcome were investigated. Data from a previous study on eDNA production determined in 24-hour biofilms of staphylococcal isolates (S*taphylococcus aureus* n=30,* Staphylococcus epidermidis* n=30) was correlated with the patients' clinical outcome after 3 and 12 months. Statistical analysis was performed using either the Spearman's rank correlations test or the t-test. eDNA production of* S. epidermidis* in 24-hour biofilms correlated with the patients' outcome ‘not cured‘ after 12 months. For* S. aureus* no such correlation was detected. Thus, eDNA may be a virulence factor of* S. epidermidis. *Quantification of eDNA production as a surrogate marker for biofilm formation might be a potential predictive marker for the management of PJI.

## 1. Introduction

Periprosthetic joint infections (PJI) are most challenging complications of orthopedic implant surgery. With the rapidly increasing number of implanted prostheses, the impact of PJI is steadily increasing. The relative incidence ranges between 2% and 2.4% of total hip (THA) and total knee arthroplasties (TKA) [1]. The pathogenesis of PJI is associated with the formation of bacterial biofilms involving the tissue around the implant and implant surfaces. Biofilm formation is a bacterial strategy to survive under adverse conditions [2]. The production of extracellular polymeric substances (EPS) protects bacteria against environmental damage. Moreover, bacteria coated by EPS are also able to escape the innate immune response [3]. Generally, biofilms with EPS production enable exchange of genes between the tightly packed bacterial cells. Moreover, their altered metabolic state leads to resistance to antibiotics and consequently persistence of infection and treatment failure [4]. About two-thirds of implant-associated infections in orthopedic surgery are caused by two staphylococcal species:* S. aureus* and* S. epidermidis *[5].

Staphylococci have different mechanisms to form biofilms, which depend on environmental conditions. The most common pathway used by* S. epidermidis* is the production of polysaccharide intercellular adhesin (PIA). PIA is actively induced through stress conditions, such as, e.g., shear flow, and heat, and enhances EPS production [6]. When bacteria were previously exposed to antibiotics, increased production of extracellular DNA (eDNA) was shown to enhance the physical properties of EPS and biofilms resistance to antibiotics [7, 8]. eDNA is released either by active secretion or by cell autolysis and was shown to be linked to the ability of bacteria to take up DNA from the environment. This feature called competence contributes to the strategy to survive in the environment. [9]. The production of eDNA is regulated by the bacterial population density in response to the accumulation of quorum sensing signals of the closely packed bacterial cells [10]. eDNA binds with other biofilm polymers (*i.e.*, polysaccharides and proteins), thus securing structural stability of the biofilm, and favors bacterial adhesion to abiotic surfaces [11]. Targeting eDNA might be a strategy for the treatment of implant-associated infections and other biofilm associated infections [7, 12, 13].

In a previous study, the time course of eDNA production in biofilms of clinical isolates of* S. aureus* and* S. epidermidis* was studied. The amount of eDNA (mean % area eDNA) was visualized and quantified using confocal laser scanning microscopy (CLSM) and TOTO™-1 staining. Image J software was used to score the images of stained biofilms.

eDNA production was greater in clinical isolates of* S. epidermidis* and* S. aureus* isolated from PJI compared to eDNA production of control isolates from the skin of healthy volunteers. After 24 hours, the amount of eDNA was greater in biofilms of* S. epidermidis* than in biofilms of* S. aureus. *The production of eDNA varies extensively during the time course of biofilm development, as well as the respective staphylococcal species [14].

The aim of the present study was to retrospectively investigate a possible association of eDNA production of in vitro biofilms of* S. aureus* and* S. epidermidis* clinical isolates from patients with PJI and the outcome of the treatment of PJI. The clinical outcomes after 3 and 12 months and the amount of eDNA production of the respective staphylococcal isolates in 24-hour biofilms were correlated. Additionally other influencing parameters like age, weight, the Charlson index for comorbidity (CCI), the site of the infection, and laboratory infection parameters including C-reactive protein, fibrinogen, and leukocyte count were studied.

## 2. Material and Methods

### 2.1. Study Design

The study population of this retrospective study was a previous study population whose pathogens, 60 clinical* S. aureus*, and* S. epidermidis* isolates from infected hip and knee prosthesis were examined for eDNA production [14].

The ethics committee of the Medical University of Vienna Austria approved the study protocol (Ethic committee no.: 19025).

### 2.2. Patient Characteristics

Patients' data were retrospectively retrieved from the electronic patient records. Information was collected and anonymously processed using the University of Vienna Research documentation and analysis platform (RDA, research documentation, and analysis). Patients' characteristics included age, weight, and body-mass-index (BMI). Comorbidities were collected and categorized using the Charlson Comorbidity Index; (Comorbidity-Adjusted Life Expectancy, CCI) [15] (Table 1). Implant indwelling time was also collected and infection classification (Table 2) was performed accordingly. Additionally inflammatory markers such as C-reactive protein (CRP), fibrinogen, and number of leucocytes were assessed at the time of diagnosis of PJI and three weeks thereafter.

The clinical outcomes after 3 and 12 months were defined as (1) cured if patients were able to walk, no further antibiotic treatment and pain medication were needed and neither local nor systemic signs of infection were present, (2) not cured, if patients continued taking antibiotics in order to cure or suppress infection or were planned for another revision surgery, or (3) deceased (Table 3). PJI were classified into early (onset < 1 month after implantation surgery), delayed (onset 3-24 months after surgery), or late infections (onset > 24 months after surgery) [16].

### 2.3. Antimicrobial Susceptibility

Antimicrobial susceptibility testing to cefoxitin, gentamicin, erythromycin, clindamycin, fusidic acid, tetracycline, fosfomycin, trimethoprim, linezolid, mupirocin, and tigecycline was performed in all staphylococcal isolates [14] using disc diffusion tests according to the protocols of European Committee on Antimicrobial Susceptibility Testing (EUCAST) (http://www.eucast.org/clinical_breakpoints/).

The multiple antibiotic resistance (MAR) index was calculated for all tested isolates according to the expression [17]

(1) $$\begin{array}{c} MAR=\frac{a}{\left(b*c\right)} \end{array}$$

where “a” is the aggregate antibiotic resistance score of all isolates, “b” is the number of antibiotics, and “c” is the number of isolates. The MAR of all tested isolates was 0,183. According to [18] a MAR index of 0.183 indicates that the aggregate antibiotic resistance is low; i.e., the isolates were in general susceptible to the tested antibiotics.

### 2.4. Statistical Methods

Spearman's rank correlation and the t-test were used to assess parallels in eDNA production, antibiotic resistance, patients clinical conditions, and outcomes. A* p*-value of <0.05 was considered to be statistically significant. eDNA values were log-transformed and checked for normal distribution before applying the t-test to calculate the approximate log-normal distribution. Calculations were performed using IBM®-SPSS® Version 24.0 (IBM Corp. Armonk. NY. USA).

## 3. Results

Sixty patients (27 male, 33 female) with a mean age of 69 year (range 17-89, median 71) were included into the study. The two age outliers, 17 and 20 year old patients, suffered from Ewing's sarcoma- or osteosarcoma and received total replacements of the femur and the knee. Thirty-eight of 60 (63.3%) patients were classified as overweight or obese with a BMI > 25 kg/m^2^: 24/60 (40%) patients were overweight and 14/60 (23.33%) were obese, (Table 1). After 12 months, the outcome in 40/48 patients was classified as cured, and the outcome in 8 patients was classified as not cured including a patient who died from the infection. A more detailed description of the outcomes with regard to pathogens or type of prosthesis is given in Table 3. A PJI considered as chronic infections were caused by* S. epidermidis.* Early or acute late infections were caused by* S. aureus* (Table 2). Twelve patients were lost during follow-up: 8 patients due to incomplete datasets and 4 patients died from their comorbidities or other age-related diseases (57-84 years; median 75 years old) during the observation period.

### 3.1. eDNA Production and Clinical Outcome

**Figure 1:**
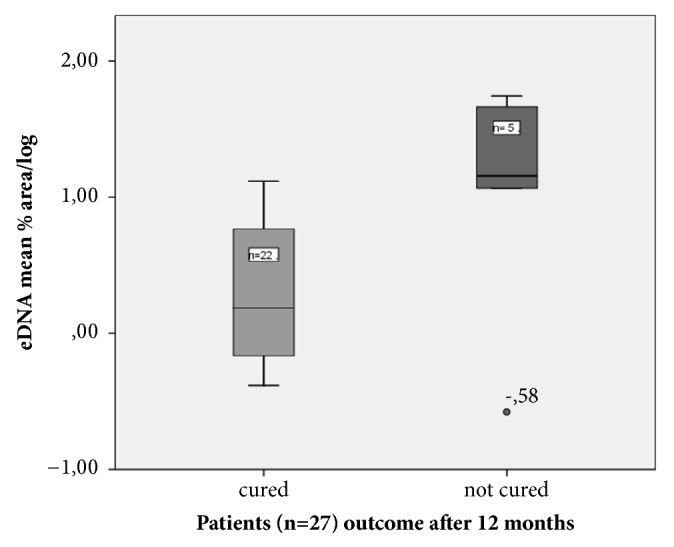
eDNA production in 24h biofilms of* S. epidermidis *is greater in isolates of patients with adverse outcome than in patients with favourable outcome.

There was a correlation between the amount of eDNA in 24 h* S. epidermidis* biofilms and patients outcome ‘not cured or respectively dead' after 12 months (n=27, r=0.391.* p*=0.044) but not for* S. aureus* (Table 4, Figure 1). For all isolates from hip prostheses, there was a positive correlation between eDNA production and the patients outcome “not cured or respectively dead” after 12 months (n=21, r=0.605, and* p*=0.004) (Table 4).

Charlson comorbidity index (CCI) showed no correlation to eDNA production of 24 hours biofilms.

### 3.2. eDNA Production and Antimicrobial Susceptibility

Among 30 clinical* S. epidermidis *isolates, 16 were methicillin-resistant* Staphylococcus epidermidis* (MRSE) and 9 and 15 showed resistance to rifampicin and clindamycin, respectively. Eleven out of 30 clinical* S. epidermidis* isolates were resistant to fusidic acid. Among 30 clinical* S. aureus* isolates, 8 were methicillin-resistant* S. aureus* (MRSA) and 1 and 5 showed resistance to rifampicin and clindamycin, respectively. One out of 30 clinical* S. aureu*s isolates was resistant to fusidic acid. A significantly lower eDNA production was only found in isolates resistant to fusidic acid: 4,86 for susceptible isolates versus 2,14 for resistant isolates [(eDNA 24h mean (% area), n=60, t=5.102,* p*<=0.001] and rifampicin: 4,76 for susceptible isolates versus 2,4 for resistant isolates [eDNA 24h mean (% area); n=60, t= 2.257, and* p*=0.028)].

### 3.3. Laboratory Parameters

The analysis of serum inflammation biomarkers revealed that all patients with* S. aureus* infections had greater mean serum levels of C-reactive protein (10.16 ± 3.33 mg/l/day; mean ± standard deviation) than patients with* S. epidermidis* infections (5.64 ± 2.14 mg/l/day) (*p*<0.001) during their 3 weeks clinical follow-up after surgery. Similarly mean fibrinogen levels were significantly greater in patients with PJI caused by* S. aureus *551.46 ± 52.51mg/l^−1^/day) than patients with PJI caused by* S. epidermidis *(457.74 (± 80.69 mg/l^−1^ /day) (*p*<0.001).

## 4. Discussion

The increasing life expectancy together with the constant progress in medicine increases the number of patients receiving medical implants, e.g., knee and hip prostheses, pacemakers, or many other medical implants and devices [19]. Therefore medical implant related infections are an increasingly substantial burden to the healthcare system [20, 21]. According to the surveillance of the European Centers for Disease Prevention and Control (ECDC) the incidence of surgical site infections (SSIs) after hip and knee surgery was 1.1%, (ranging from 0.3% to 3.8%) for THA and 0.6% (range 0.0% to 3.4%) for TKA. http://ecdc.europa.eu/en/healthtopics/Healthcare-associated_infections/surgical-site-infections/Pages/Annual-epidemiological-report-2016.aspx. In order to treat these infections a thorough understanding of the pathogenesis and the pathogens is pivotal.Clinical outcomes of PJI with respect to their causing pathogen and respective biofilm formation ability are subject of a few studies only. A prospective study in 124 patients with orthopedic implant-related osteomyelitis showed the influence of biofilm formation and antibiotic resistance on the outcome. In the subgroup of 90 patients with lower extremity infections the increase of* S. epidermidis *biofilm thickness correlated with decreased cure rates [18]. Mittag et al. examined clinical outcomes after infected knee and hip arthroplasty using clinical data of 64 patients and scores including the Western Ontario and McMaster Universities- (WOMAC-) Index, the Harris Hip Score (HHS) and the Hospital for Special Surgery Score (HSS). They did not demonstrate a correlation between implant infection classified according to the modified Tsukayama classification system [22] and outcome defined using WOMAC, HSS or HHS score [23]. However, in this study, the most frequent pathogens were* Enterococcus spp*. followed by a mixture of bacteria causing polymicrobial infections

So far a correlation between eDNA production in staphylococcal biofilms and clinical outcome of PJI has not been reported in the literature. In the present study* S. epidermidis* isolates showed significantly greater eDNA production than* S. aureus* isolates in the respective 24h biofilms [14]. Infections of* S. aureus* and* S. epidermidis* are considered distinguishable by their clinical symptoms and course:* S. aureus* infections usually present with classical local signs and symptoms of infection with pain, redness, swelling, temperature and impaired function and a systemic immune response with fever, hypotension, etc., leucocytosis and elevated C-reactive protein, etc... Infection caused by* S. epidermidis *presents usually with subacute signs and symptoms of infection and an unspecific and delayed onset. In the present patient population infections with* S. epidermidis* presented as chronic infections. Early or late acute infections were exclusively caused by* S. aureus* (Table 2).We were able to demonstrate that eDNA production of* S. epidermidis *24 hours biofilms correlated with the clinical outcome ‘not cured respectively dead' after 12 months, (p=0.044). eDNA production is a relatively stable characteristic of many* S. epidermidis* strains [14]. Thus, it may be hypothesized that production of eDNA by* S. epidermidis* isolated from PJI contributes to the pathogenesis and may be used to predict clinical outcome.

Exposure to antibiotics has been linked to eDNA production in biofilms [24, 25]. Perioperative antibiotic prophylaxis is a standard of care in orthopaedic prosthetic surgery [26]. However, Doroshenko et al. reported higher eDNA levels in biofilms of* S. epidermidis *after prior exposure to vancomycin [25]. Schilcher et al. described that subinhibitory concentrations of clindamycin increased the ability of* S. aureus *to form biofilms and shift the composition of the biofilm matrix towards higher eDNA content [27]. In the present study, isolates resistant to rifampicin and fucidic acid produced less amounts of eDNA than susceptible ones. But, antimicrobial resistance was tested only using the disk diffusion method testing planktonic bacteria compared to biofilm susceptibility testing performed in the other studies [27] or as demonstrated by Brady and colleagues in their study comparing minimum biofilm eradication concentration and minimum inhibitory concentration breakpoint in planktonic versus biofilm grown staphylococci [28]. However, further investigation into the effects of rifampicin or fusidic acid on eDNA production should be done performing resistance testing in biofilm growth systems.

Inflammation biomarkers such as fibrinogen, C-reactive protein, and leucocyte count did not correlate with eDNA levels of 24 hours biofilms of the respective pathogens. Yet, a significant difference between the clinical presentation of PJI caused by either* S. aureus* or* S. epidermidis* was found in our patient population likewise in earlier studies [29, 30], where patients with PJI caused by* S. aureus* exhibited greater serum levels of C-reactive protein and fibrinogen compared to patients with PJI caused by* S. epidermidis*.

The limitations of the present study are inherent to the retrospective nature of the study because not all clinical and laboratory data are available, and there is a rather small sample size of a nevertheless very well defined patient population. Due to the small sample size, multivariate statistical analysis was not indicated. Moreover, in vitro conditions of biofilm formation may not fully reflect clinical biofilms in PJI [31].

## 5. Conclusion

In conclusion, a correlation between increased eDNA production of* S. epidermidis* 24h biofilms and adverse clinical outcome after 12 months was demonstrated. Quantification of eDNA production of the pathogen as a surrogate marker for biofilm formation might be a potential predictive marker for the management of PJI caused by* S. epidermidis*. eDNA might also be a possible therapeutic target. Further prospective and sufficiently powered clinical studies will be needed to strengthen the role of eDNA production of pathogens on the clinical course and its relevance in PJI.

## Figures and Tables

**Table 1:** Patient's demographic data and health index.

Patients description	n=60
Age	
17-89 years	mean 69
Sex	
female	33 (55%)
male	27 (45%)
BMI (kg/m2)	
underweight (< 19)	2 (3,3%)
Normal (19 - < 25)	19 (31%)
overweight (25 - < 30)	24 (40%)
obesity (>30)	14 (3,3%)
Comorbidities-Charlson Index	
0	8 (13,3%)
1	16 (26,7%)
2	19 (31,7 %)
3	5 (8,3 %)
4	4 (6,7 %)
5	2 (3,3%)
6	3 (5%)
7	2 (3,3%)
12	1 (1,7%)

**Table 2:** Characteristics of implant infect classification with regard to bacterial species or explanted joint.

	Microorganism		Joint	
	*S. aureus*	*S. epidermidis*	hip	knee
	n = 30	n = 30	n = 29	n = 31
Implant classification				

primary	23 (76.7 %)	13 (43.3%)	16 (53.3 %)	20 (66.7 %)
secondary (>2)	7 (23.3 %)	17 (56.7%)	14 (46.7 %)	10 (33.3 %)

Infect classification				

early	12 (40 %)	-	5 (16.7 %)	7 (22.6 %)
late	18 (60 %)	-	11 (36.7 %)	8 (26.7 %)
chronic	-	30 (100%)	14 (46.7 %)	15 (50 %)

**Table 3:** Outcome classified with regard to bacterial species or explanted joint type.

	Microorganism		Joint	
	*S. aureus*	*S. epidermidis*	hip	knee
Outcome after **3 months**	n=25	n=28	n=23	n=30

cured	16 (64%)	22 (79%)	17 (74%)	21 (70%)
not cured	6 (24%)	5 (18%)	3 (13%)	8 (27%)
dead	3 (12%)	1 (4%)	3 (13%)	1 (3%)

Outcome after **12 months**	n=21	n=27	n=21	n=27

cured	18 (86%)	22 (81%)	18 (86%)	22 (81%)
not cured	3 (14%)	4 (15%)	2 (10%)	5 (19%)
dead	0 (0%)	1 (4%)	1 (5%)	0 (0%)

**Table 4:** eDNA production after 24 h in correlation to outcomes after 3 and 12 months with regard to bacterial species or explanted joint.

	*S. aureus*	*S. epidermidis*	Hip isolates	Knee isolates
	eDNA 24log	eDNA 24log	eDNA24log	eDNA24log
Outcome after **3 months**	n=25	n=28	n=23	n=30

Correlation Coefficient	-0.028	-0.161	-0.293	-0.013
Sig. (2-tailed)	0.894	0.413	0.175	0.945

Outcome after **12 months**	n=21	n=27	n=21	n=27

Correlation Coefficient	-0.022	0.391	0.605	-0.018
Sig. (2-tailed)	0.923	0.044^**∗**^	0.004^**∗∗**^	0.928

## Data Availability

The data used to support the findings of this study are available from the corresponding author upon request.

## Supplementary Materials

Supplementary Materials Supplementary material contains detailed data of the eDNA production after 6 and 24 hours in biofilms of bacterial isolates from hip and knee joint after explantation. The measurements were done using CLSM and TOTO1 staining. Table S1: eDNA production (mean % area eDNA) after 6 and 24 hours in biofilms of hip and knee joint explants irrespective of staphylococcal species.
