# Supplementary material for: Bacterial Extracellular DNA Production Is Associated with Outcome of Prosthetic Joint Infections
Source: Biomed Res Int. 2018 Oct 22;2018:1067413. doi: 10.1155/2018/1067413 (PMC6217901; doi:10.1155/2018/1067413)
Supplement: Supplementary Materials — Supplementary material contains detailed data of the eDNA production after 6 and 24 hours in biofilms of bacterial isolates from hip and knee joint after explantation. The measurements were done using CLSM and TOTO1 staining. Table S1: eDNA production (mean % area eDNA) after 6 and 24 hours in biofilms of hip and knee joint explants irrespective of staphylococcal species. [file 1067413.f1.doc]

**Supplemental material
eDNA production (mean % area eDNA) in hip and knee joint explants respectively.**

|  | CLSM mean % area eDNA | | | |
| --- | --- | --- | --- | --- |
|  | Min. | | Max. | Mean (SD) |
| **Hip (n=31)** |  | |  |  |
| eDNA 6h | 0.12 | | 4,55 | 2.16 ± 1.4 |
| eDNA 24h | 0.24 | | 55,11 | 7.79 ± 13.52 |
|  |  | |  |  |
| **Knee (n=29)** | |  | |  |
| eDNA 6h | 0.19 | | 4,23 | 1.53 ± 1.17 |
| eDNA 24h | 0.11 | | 13,10 | 2.02 ± 3.1 |
